# Supplementary material for: Extracellular release of two peptidases dominates generation of the trypanosome quorum-sensing signal
Source: Nat Commun. 2022 Jun 9;13:3322. doi: 10.1038/s41467-022-31057-1 (PMC9184580; doi:10.1038/s41467-022-31057-1)
Supplement: Supplementary file 1 — Supplementary Information [file 41467_2022_31057_MOESM1_ESM.pdf]

Extracellular release of two peptidases dominates generation of the trypanosome quorum-sensing signal

Mabel Deladem Tettey, Federico Rojas and Keith R. Matthews

Supplementary information

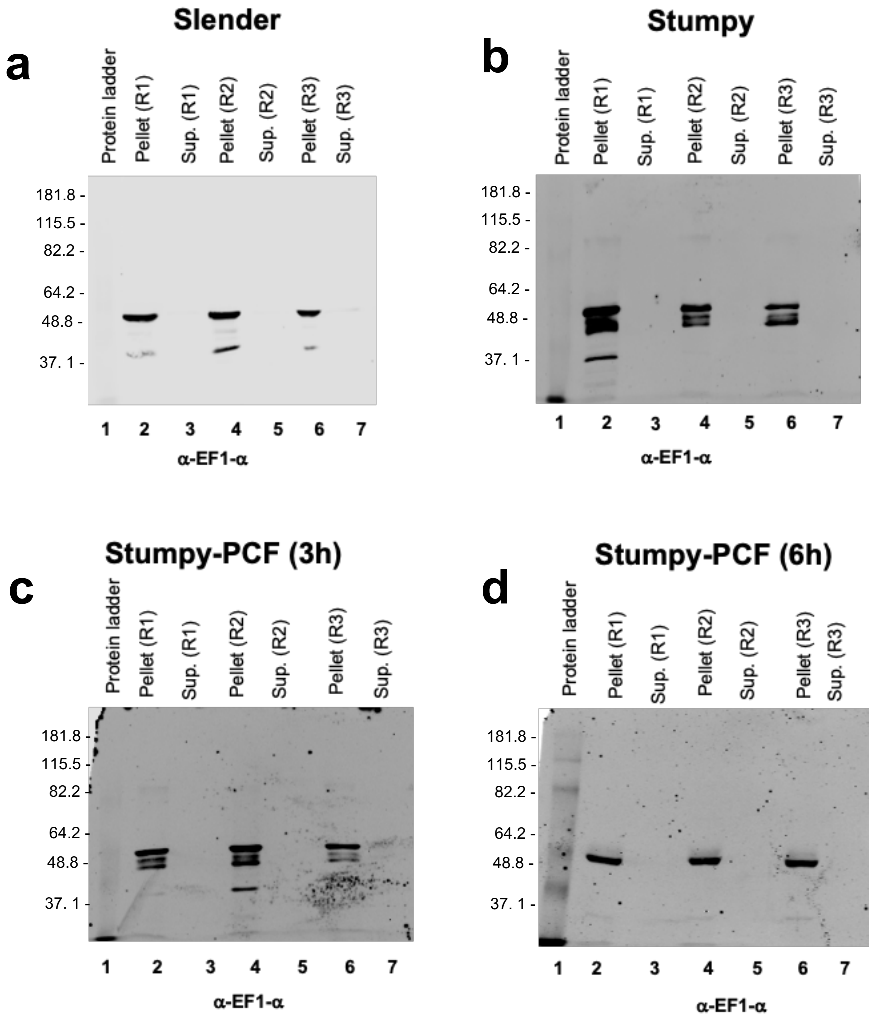

e

Gene IDs	Protein description	Log_{10} (Average Normalised abundance)		
		Stumpy	3hr	6hr
Tb927.11.2500	Metallocarboxypeptidase 1	8.21	6.79	6.57
Tb927.8.8330	Calpain	7.08	4.62	4.53
Tb927.3.2090	Aminopeptidase P1	5.88	5.41	5.04
Tb927.8.7020	Peptidase 1	6.57	4.73	5.20
Tb927.11.6590	Aminopeptidase 2	8.02	6.83	6.31
Tb927.6.400	Peptidase M20/M25/M40	7.56	6.63	6.22
Tb927.11.3570	Aminopeptidase 1	7.68	6.83	6.17
Tb927.3.3410	Aspartyl aminopeptidase	6.84	6.36	5.78
Tb927.10.12260	Cytosolic nonspecific dipeptidase	0.00	6.88	6.35
Tb927.11.12850	Oligopeptidase B	6.75	5.64	5.13
Tb927.3.4750	Aminopeptidase 3	6.54	6.06	5.61
Tb927.1.2100	Calpain-like cysteine peptidase	0.00	4.59	3.79

Supplementary Figure 1

Supplementary Figure 1 : Analysis of trypanosome-released proteins identified many peptidases

a. Slender supernatant, b. stumpy supernatant, c. Stumpy-procyclic differentiating cells supernatant (1h in HMI-9, 2h in CMM), d. Stumpy-procyclic differentiating cells supernatant (4h in HMI-9, 2h in CMM). Three biological replicates are shown in each case, with EF1-alpha signal being detected in the cell pellet but not cell supernatant fractions for each, reflecting the cell integrity of the material used to analyse the secreted/release material from each parasite sample. These samples were used for mass spectrometry analysis. Molecular weight markers in kDa. Panel e shows peptidases identified in the study.

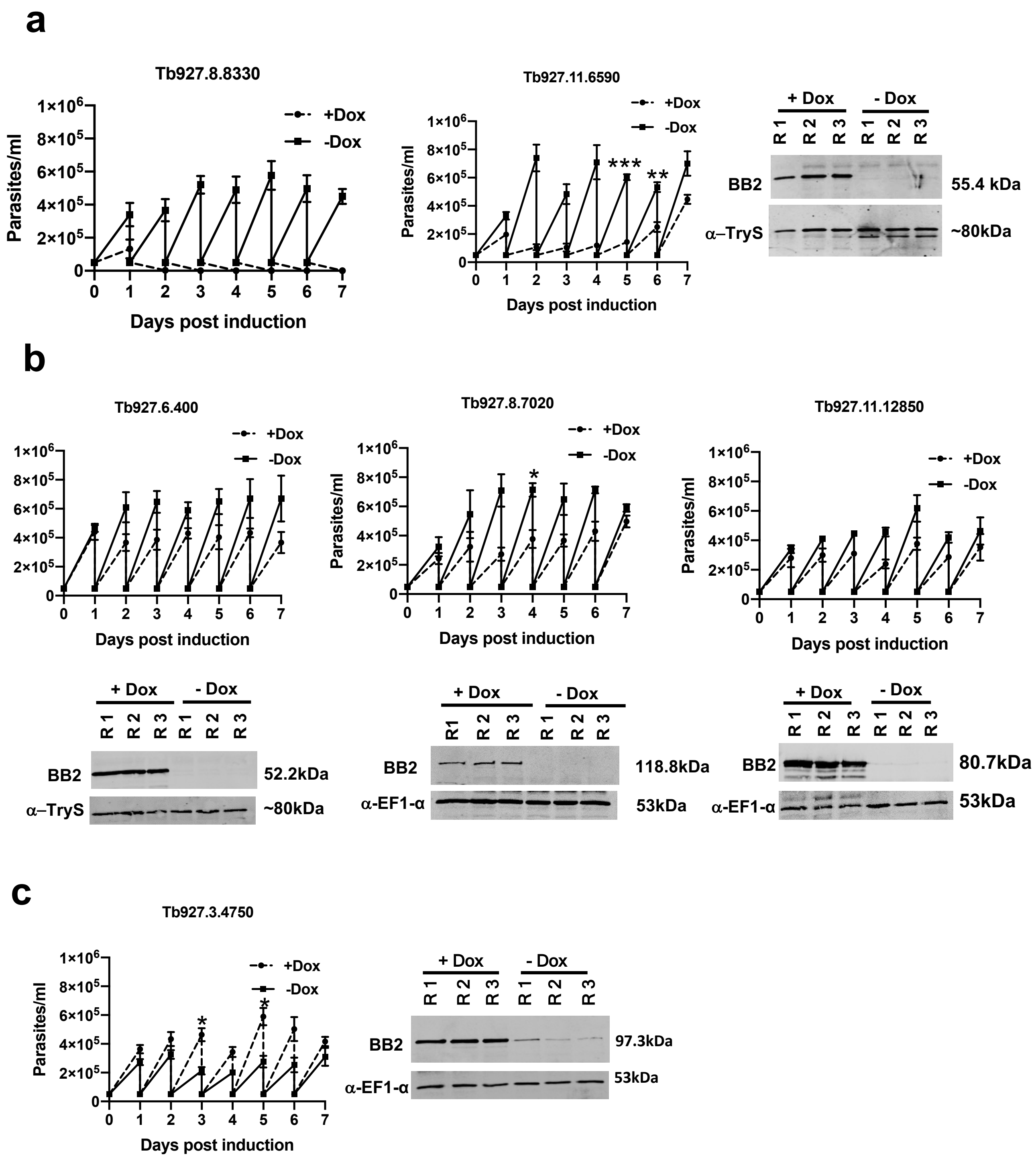

Supplementary Figure 2a, b, c

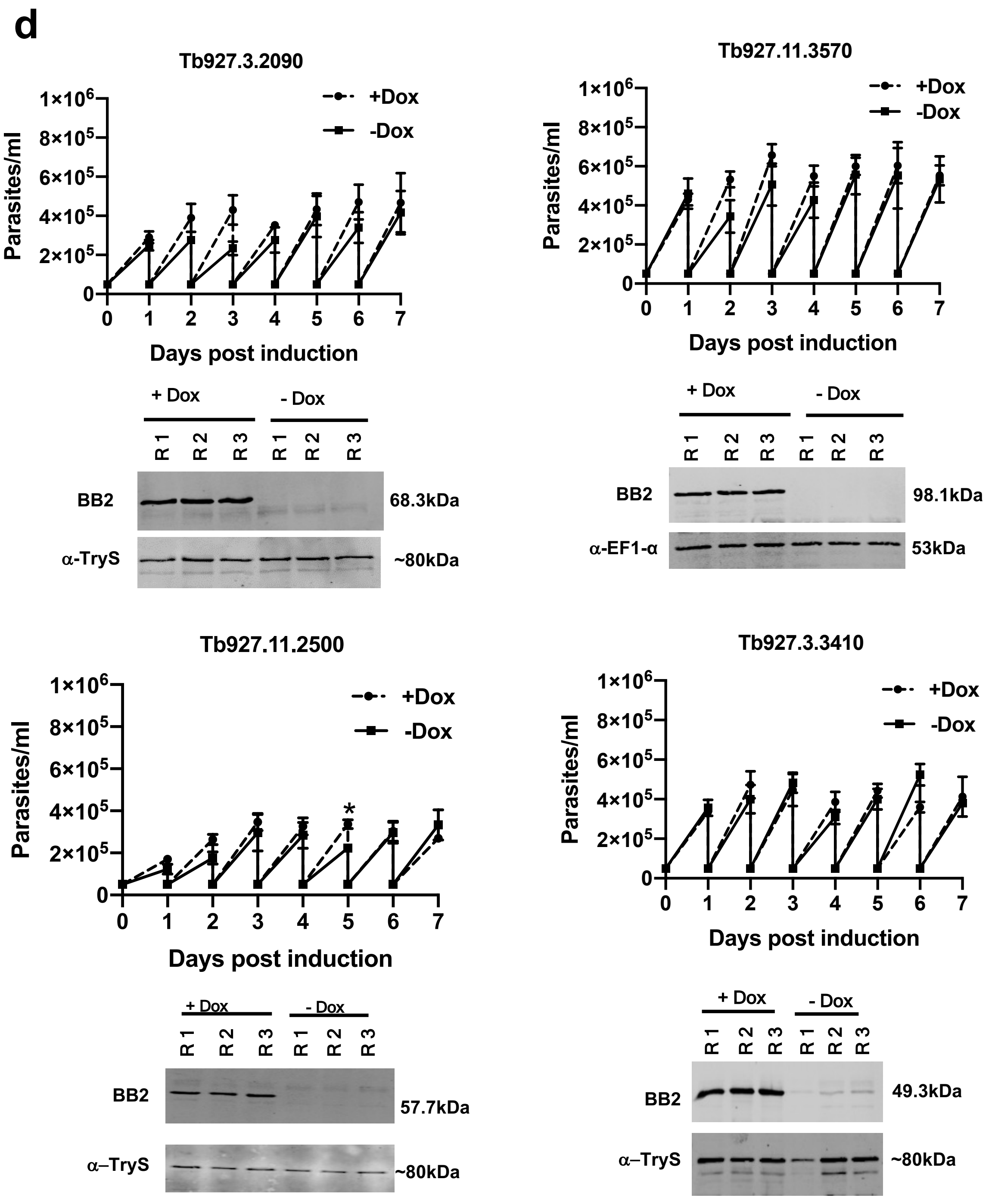
d
Tb927.3.2090
Tb927.11.3570
Tb927.11.2500
Tb927.3.3410
Parasites/ml
Days post induction
+Dox
-Dox
+ Dox
- Dox
R1 R2 R3 R1 R2 R3
BB2
α-TryS
α-EF1-α
68.3kDa
~80kDa
98.1kDa
53kDa
57.7kDa
49.3kDa

Supplementary Figure 2 : ***In vitro* growth profile of parasite cell lines induced, or not, to ectopically overexpress each peptidase**

Growth profile of parasites induced (dotted lines), or not (solid lines), to ectopically overexpress each peptidase. On each day, parasites were diluted to $1x10^5$ parasites per ml; each growth profile represents the mean and standard deviation of three biological replicates. The accompanying panels demonstrate the inducible expression of each peptidase detected using the Ty1-eptitope tag specific BB2 antibody. Loading is shown by the abundance of EF1-alpha in each sample. No accompanying western blot is provided for Tb927.8.8330 because the rapid cell death observed after expression prevented the isolation of cellular proteins. a. Peptidases whose expression strongly limits growth of the parasites b. Peptidases whose expression reduces growth of the parasites moderately c. Peptidase whose expression enhances growth of the parasites d. Peptidases that have little discernible effect on the growth of the parasites when ectopically expressed. Two-way ANOVA analysis was performed followed by Šídák's multiple comparison test.

$p<0.05$ (*); $p<0.002$ (**), $p<0.0002$ (***). Tb927.11.6590; $p=0.0002$ and 0.0080 respectively, Tb927.8.7020; $p=0.1523$, Tb927.3.4750; $p= 0.0413$ and 0.0386 respectively, Tb927.11.2500; $p= 0.0311$.

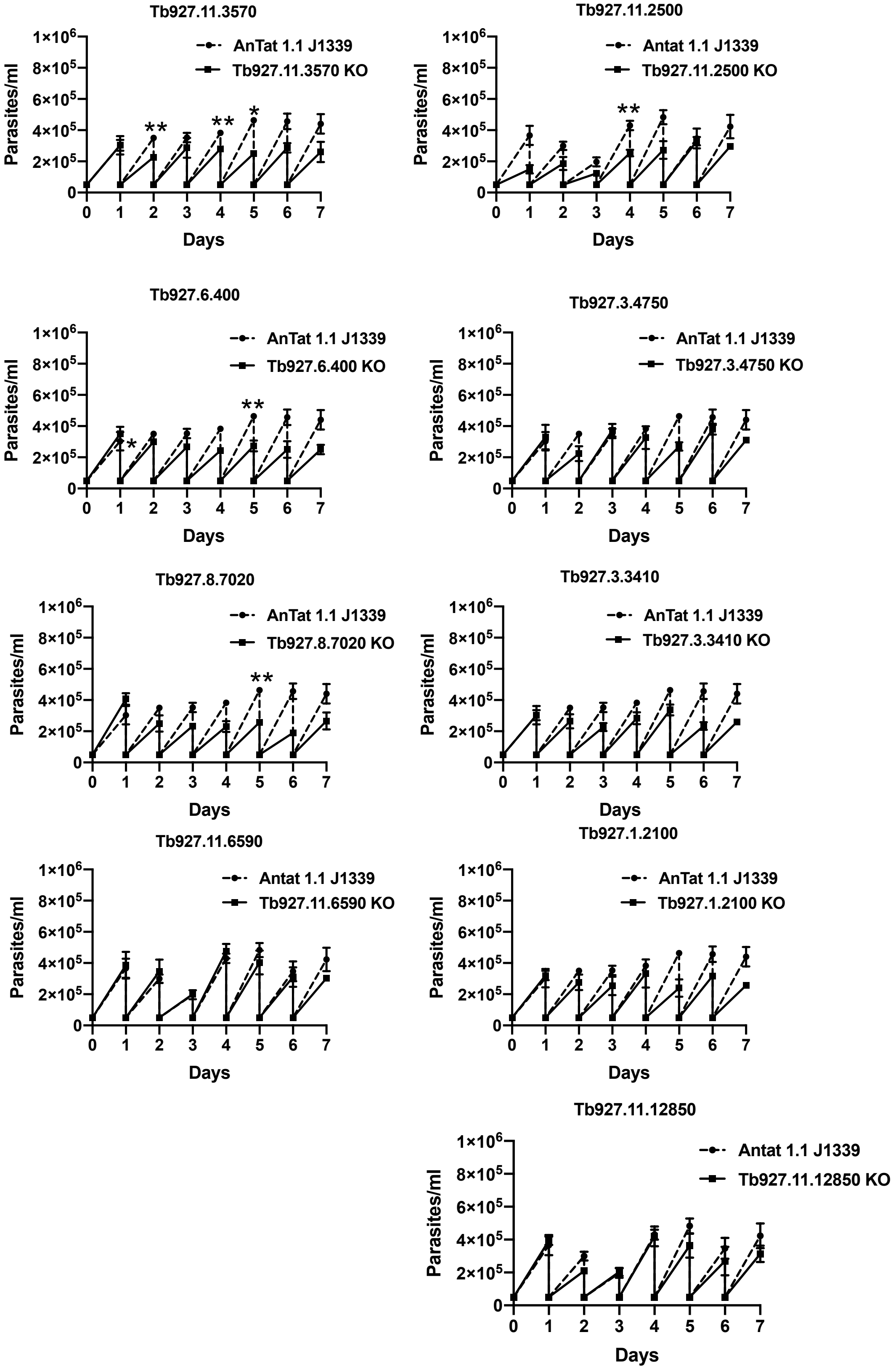

Supplementary Figure 3 : ***In vitro* growth of null mutant parasite lines for each identified peptidase.**

In vitro growth profile of each peptidase null mutant line versus growth of parental cell line (*T. brucei* AnTat1.1 J1339). Each day parasites were diluted to $1x10^5$ parasites/ml. Each growth profile represents the mean and standard deviation of three biological replicates. Two-way ANOVA test was performed followed by Šídák's multiple comparison test. $p<0.05$ (*); $p<0.002$ (**). Tb927.11.3570; p=0.0024, 0.0047 and 0.124 respectively, Tb927.11.2500; p= 0.0250, Tb927.6.400; p=0.0495 and 0.0017 respectively, Tb927.8.7020; p= 0.0077

KO growth in vivo

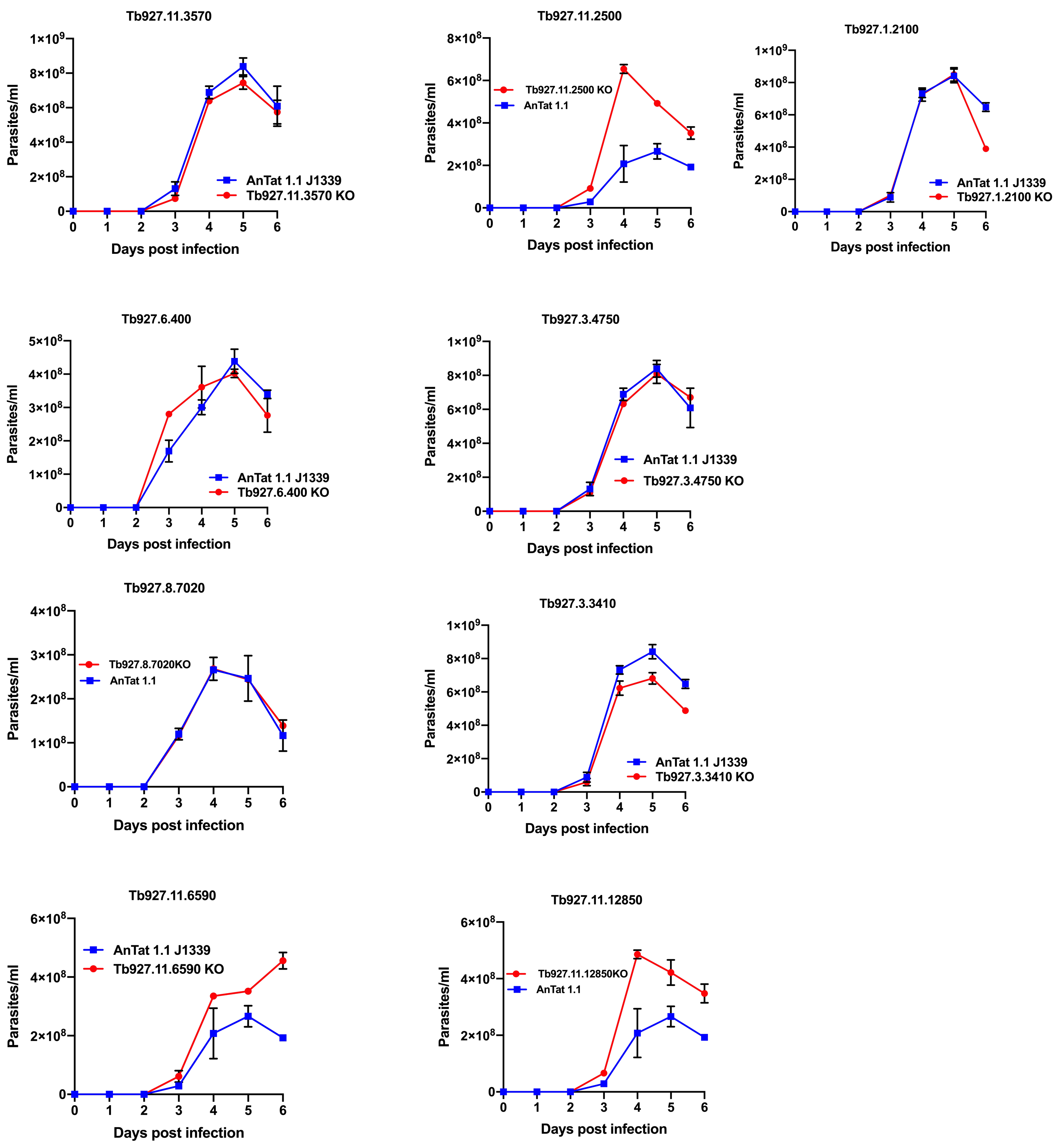

Supplementary Figure 4 : ***In vivo* growth of null mutant parasite lines for each identified peptidase.**
In vivo growth profile (mean ±SEM) of each peptidase null mutant line (red lines) versus growth of the parental cell line (*T. brucei* AnTat1.1 J1339) (blue lines). In each case two mice were infected with the null mutant line and two with the parental line and infections carried out in parallel.

*Tb*MCP1 addback

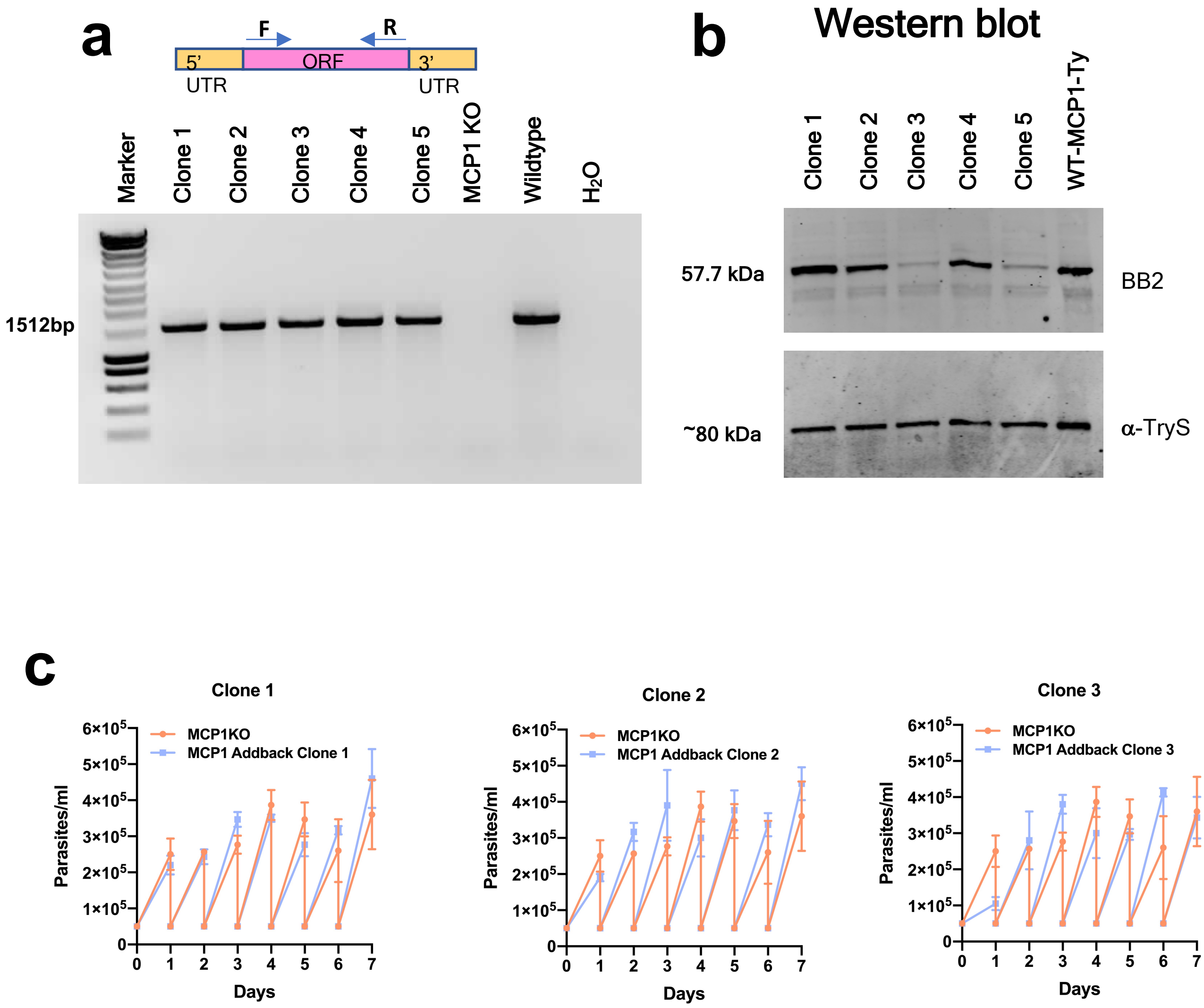

Supplementary Figure 5 : **Validation of MCP1 add-back expression and growth profile *in vitro***

a. PCR analysis of Tb927.11.2500 null mutants with a restored, add back, gene copy. Gene specific primers amplify the Tb927.11.2500 gene in the add back lines and parental cells, but not in the null mutant.

b. Western blot analysis confirming expression of the add back copy of Tb927.11.2500. The control line is parental parasites with an endogenously Ty1 epitope tagged copy of Tb927.11.2500 (WT-MCP1-ty). Equivalent loading is shown by trypanothione synthetase (TryS).

c. *In vitro* growth profile (mean ±SEM) of three add back clones in comparison to the null mutant. In each the growth *in vitro* of the cell lines was equivalent. n=3 per cell line.

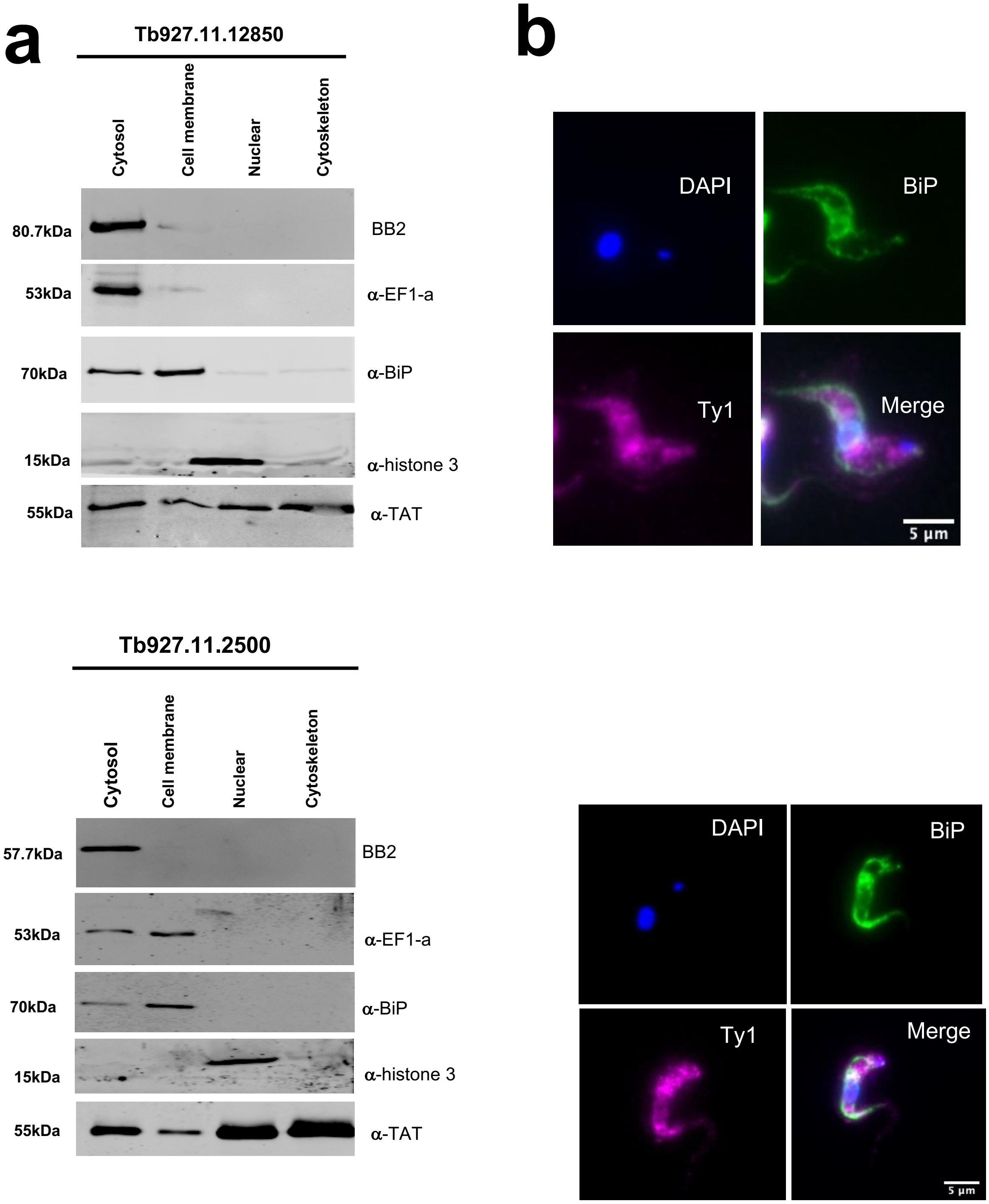

Supplementary Figure 6 : **Oligopeptidase B and metallocarboxypeptidase 1 are cytosolic proteins**

a. Cell fractionation of parasites expressing endogenously Ty1 epitope tagged Tb927.11.12850 and Tb927.11.2500. The tagged proteins ('BB2') were mostly cytosolically associated. Marker proteins were EF1-alpha (cytosol and some membrane fraction), BiP (some cytosolic and mainly ER), histone H3 (nuclear) and alpha tubulin (detected in all fractions).

b. Immunofluorescence analysis of the distribution of endogenously Ty1 epitope tagged Tb927.11.12850 or Tb927.11.2500 with respect to the ER marker BiP. While BiP exhibited the expected reticulated pattern, the peptidases were uniformly distributed throughout the cell, reflective of cytosolic location.

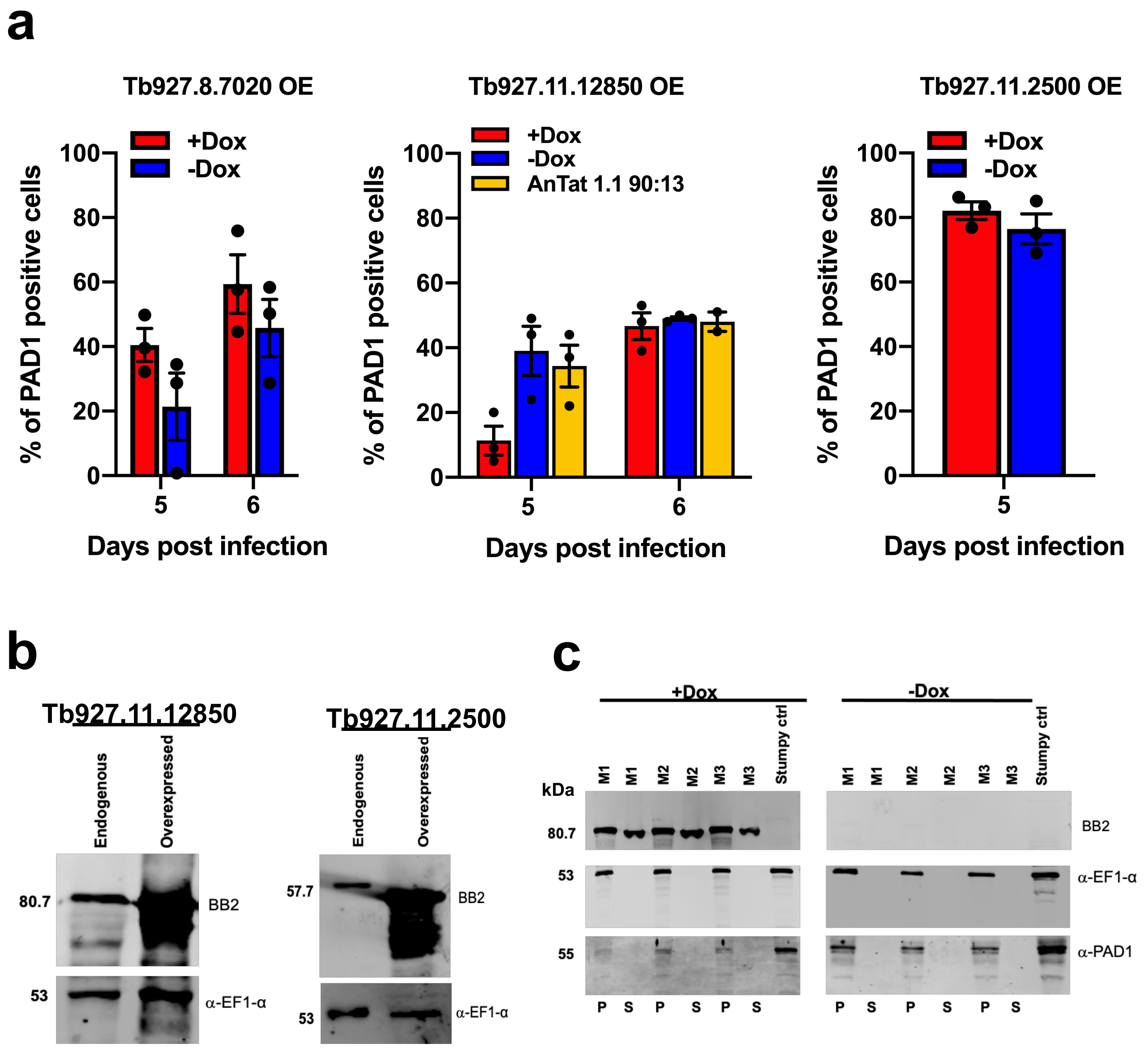

Supplementary Figure 7

a. PAD1 scoring (mean ±SEM) for the three peptidases whose ectopic overexpression enhanced stumpy differentiation. n=3 per cell line.
b. Comparison of the expression of endogenously tagged Tb927.11.12850 and Tb927.11.2500 and their respective ectopic overexpression. The level of expression was quantified using *ImageJ*. Molecular weight markers in kDa.
c. Comparison of the level of PAD1 expression in Tb927.11.12850 OE/RBP7 KO cells and a bona fide stumpy cells. Molecular weight markers in kDa.

Supplementary figure 7

Double peptidase knockout	
	Tb927.11.12850 KO vs double gene KO
Day 4	* (p=0.0309)
Day 5	**** (p<0.0001)
Day 6	* (p=0.0133)
Day 7	** (p=0.005)
Double peptidase knockout + OPB add back	
	TbOPBKO::TbMCP1KO vs TbOPBKO::TbMCP1KO ::TbOPB AB
Day 4	**** (p<0.0001)
Day 5	**** (p<0.0001)
Day 6	** (p=0.0025)
Day 7	* (p=0.0172)

Supplementary Table 1: Statistical analysis for mice infection in Figure 6. ns: not significant, p-value > 0.05, p-value < 0.05 (*), p-value < 0.002 (**), p-value < 0.0002 (***), p-value < 0.0001 (****). . Two-way ANOVA analysis followed by Tukey's comparison test were performed.

Supplementary Table 2: Primers for endogenous tagging of the peptidases (CRISPR/Cas9)

G00	
AAAAGCACCGACTCGGTGCCACTTTTTCAAGTTGATAACGGACTAGCCTTATTTTAACTTGCTATTTCTAGCTCTAAAAC	
Tb927.8.7020 C-terminal tagging	
Upstream forward primer	AACCTCCCTGTCATTCGTTGCAGGACCTTC GAGGTACATACAAA
Upstream reverse primer	GCTGTCACAAAAAAAACAACAAACAAACCTccaatttgagagacctgtgc
3' sgRNA primer	gaaattaatacgactcactataggGTGTATTGAACGTGGTGGGTgttttagagctagaaatagc
Tb927.11.2500 N-terminal tagging	
Upstream forward primer	TCGTATTTGACCGGTAACCAACACAAGTAAgtataatgcagacctgctgcaa
Upstream reverse primer	GACGCGCTCGAGCTCTTTGTATGCCTTCAT ATCCAAGGGATCTTGATTGGT
5' sgRNA primer	gaaattaatacgactcactataggAATAAAGAATACAAAAGATAgttttagagctagaaatagc
Tb927.8.8330 C-terminal tagging	
Upstream forward primer	TTTGCCTTATCGGATGATAGCGACACTGAC GAGGTACATACAAA
Upstream reverse primer	TTCAGACCAATAATTAATATACCACAACCAccaatttgagagacctgtgc
3' sgRNA primer	gaaattaatacgactcactataggTATATATGTCTGATAGTAAGgttttagagctagaaatagc
Tb927.6.400 C-terminal tagging	
Upstream forward primer	TACCATGCAACGCCAAAGATACATAAGCAG GAGGTACATACAAA
Upstream reverse primer	GCACGTTTAACCAACAGGACCGTAAATGCAccaatttgagagacctgtgc
3' sgRNA primer	gaaattaatacgactcactataggGGGGAGCAAACTTGAAGCAAgttttagagctagaaatagc
Tb927.11.3570 N-terminal tagging	
Upstream forward primer	AGAAGGATCACACATATTTGTTGGGCACCGgtataatgcagacctgctgc
Upstream reverse primer	TGGGAGAGTATCCCGTTCGATCGTGGACATATCCAAGGGATCTTGATTGGT
5' sgRNA primer	gaaattaatacgactcactataggAGCGTATGCTCCTATTCTTGgttttagagctagaaatagc
Tb927.11.6590 C-terminal tagging	
Upstream forward primer	GCCTTACTTACCGAGTACTTCCGAAAATTG GAGGTACATACAAA
Upstream reverse primer	CCTACAACGATTTTCCAAATTCGGGAACCGccaatttgagagacctgtgc
3' sgRNA primer	gaaattaatacgactcactataggGCCACATGGCGTGCAAGTATgttttagagctagaaatagc
Tb927.3.2090 N-terminal tagging	
Upstream forward primer	TACGACACACTCTTTTTACGGTGGCCGCTGgtataatgcagacctgctgcaa
Upstream reverse primer	AGAAAGAATCTTGGCACTGTTGGTCGACAT ATCCAAGGGATCTTGATTGGT
5' sgRNA primer	gaaattaatacgactcactataggTGTCTATCAAAGGTGAAATAgttttagagctagaaatagc
Tb927.11.12850 C-terminal tagging	
Downstream forward primer	CATCTCAATGTACGGCAGCTGCTGCGGAAG GAGGTACATACAAA
Downstream reverse primer	TTCTACCTCATTTTCTGTGAAACACATCCTccaatttgagagacctgtgc
3' sgRNA primer	gaaattaatacgactcactataggGAAACTTGTGGAGCTAAGGTgttttagagctagaaatagc
Tb927.3.4750 C-terminal tagging	
Downstream forward primer	CAAAAGCTCTCCTACCTGTTCTTTCCGCGG GAGGTACATACAAA
Downstream reverse primer	TGTTTCCTGCACACACAGTCATGCGTAGTGccaatttgagagacctgtgc
3' sgRNA primer	gaaattaatacgactcactataggGAGCCATGCACAAAATATACgttttagagctagaaatagc
Tb927.1.2100 C-terminal tagging	
Downstream forward primer	CCTGAGCTGAAGGTGGACTCCTCCGTGTGC GAGGTACATACAAA
Downstream reverse primer	TGGGATTAAAAAAAATGGGTGGGAATCTGAccaatttgagagacctgtgc
3' sgRNA primer	gaaattaatacgactcactataggCAGTAGCGGTAAGCAAAAGAgttttagagctagaaatagc
Tb927.10.12260 C-terminal tagging	
Downstream forward primer	TTTCACGCAACGCCAAAGTTGCGAAAGGAT GAGGTACATACAAA
Downstream reverse primer	TTGAAAGGTAAATTATAAACAAACGCTCCGccaatttgagagacctgtgc
3' sgRNA primer	gaaattaatacgactcactataggACGCTACCTCTCTCCTTCACgttttagagctagaaatagc

Supplementary Table 3: Primers for gene deletion (CRISPR-Cas9)

Tb927.8.7020 knockout	
Upstream forward primer	GTCAAAACATGAACACATGCACGCGCACCAgtataatgcagacctgctgc
5' sgRNA primer	gaaattaatacgactcactataggTTGTTAAATACGCGATATGCgttttagagctagaaatagc
Downstream reverse KO primer	GCTGTCACAAAAAAAACAACAAACAAACCTccggaaccactaccagaacc
3' sgRNA primer	gaaattaatacgactcactataggGTGTATTGAACGTGGTGGGTgttttagagctagaaatagc
Tb927.11.2500 knockout	
Upstream forward primer	TCGTATTTGACCGGTAACCAACACAAGTAAgtataatgcagacctgctgcaa
5' sgRNA primer	gaaattaatacgactcactataggAATAAAGAATACAAAAGATAgttttagagctagaaatagc
Downstream reverse KO primer	GATAGATCAAGATGACCAACCATTCATACAccggaaccactaccagaacc
3' sgRNA primer	gaaattaatacgactcactataggGAATGGAAAGGGGTAACGACgttttagagctagaaatagc
Tb927.6.400 knockout	
Upstream forward primer	ACTAACCATACAAGCATTCGAATTTTCAGTgtataatgcagacctgctgc
5' sgRNA primer	gaaattaatacgactcactataggAAGAGCGCAAACTTGTGAATgttttagagctagaaatagc
Downstream reverse KO primer	GCACGTTTAACCAACAGGACCGTAAATGCAccggaaccactaccagaacc
3' sgRNA primer	gaaattaatacgactcactataggGGGGAGCAAACTTGAAGCAAgttttagagctagaaatagc
Tb927.11.3570 knockout	
Upstream forward primer	AGAAGGATCACACATATTTGTTGGGCACCGgtataatgcagacctgctgc
5' sgRNA primer	gaaattaatacgactcactataggAGCGTATGCTCCTATTCTTGgttttagagctagaaatagc
Downstream reverse KO primer	CAGCTGCATCCACTAATTTAGCAACAGCCAccggaaccactaccagaacc
3' sgRNA primer	gaaattaatacgactcactataggTACGTTATTACCATTAGAATgttttagagctagaaatagc
Tb927.11.6590 knockout	
Upstream forward primer	ACAAAGGGAGGGAGGAAAGAAACGCACGCAgtataatgcagacctgctgc
5' sgRNA primer	gaaattaatacgactcactataggAAACAAGTCAATAAATAAGAgttttagagctagaaatagc
Downstream reverse KO primer	CCTACAACGATTTTCCAAATTCGGGAACCGccggaaccactaccagaacc
3' sgRNA primer	gaaattaatacgactcactataggGCCACATGGCGTGCAAGTATgttttagagctagaaatagc
Tb927.3.3410 knockout	
Upstream forward primer	GTTGCTGATACAGTGTGTATCGAGCATTTTgtataatgcagacctgctgc
5' sgRNA primer	gaaattaatacgactcactataggTCATTACCATTCCTTTCCCGgttttagagctagaaatagc
Downstream reverse KO primer	ACTTGATATTATTTATAAATAACCCCAATAccggaaccactaccagaacc
3' sgRNA primer	gaaattaatacgactcactataggTCTACTTCGCTAACTGGAGTgttttagagctagaaatagc
Tb927.11.12850 knockout	
Upstream forward primer	ATCCGATTGCTGGGCTCCCTCAACACAATTgtataatgcagacctgctgc
5' sgRNA primer	gaaattaatacgactcactataggGTATACACTTGCACACTCGGgttttagagctagaaatagc
Downstream reverse KO primer	TTCTACCTCATTTTCTGTGAAACACATCCTccggaaccactaccagaacc
3' sgRNA primer	gaaattaatacgactcactataggGAAACTTGTGGAGCTAAGGTgttttagagctagaaatagc
Tb927.3.4750 knockout	
Upstream forward primer	CAACCTTGCGCTTTCTCACTTTTCAGACAAgtataatgcagacctgctgc
5' sgRNA primer	gaaattaatacgactcactataggGTTAACAAGTTATCAATCAAgttttagagctagaaatagc
Downstream reverse KO primer	TGTTTCCTGCACACACAGTCATGCGTAGTGccggaaccactaccagaacc
3' sgRNA primer	gaaattaatacgactcactataggGAGCCATGCACAAAATATACgttttagagctagaaatagc
Tb927.1.2100 knockout	
Upstream forward primer	TTATTTGAGAATAAGCGAATCCAAGCATATgtataatgcagacctgctgc
5' sgRNA primer	gaaattaatacgactcactataggCTCTTATCCTTAAGCATCAGgttttagagctagaaatagc
Downstream reverse KO primer	TGGGATTAAAAAAAATGGGTGGGAATCTGAccggaaccactaccagaacc
3' sgRNA primer	gaaattaatacgactcactataggCAGTAGCGGTAAGCAAAAGAgttttagagctagaaatagc

Supplementary Table 4: Primer sequences for overexpression (Spe1, BamH1)

Tb927.3.2090 over-expression	
Forward primer	ACTAGTATGTCGACCAACAGTGCCAA
Reverse Primer	GGATCCTCAACAACCGCCCTCAA
Tb927.11.2500 over-expression	
Forward primer	ACTAGTATGAAGGCATACAAA
Reverse Primer	GGATCCTCAGTTGGCATCGTCACGGT
Tb927.8.8330 over-expression	
Forward primer	ACTAGTATGGGTTGTGGTGGATCAAA
Reverse Primer	GGATCCCTAGTCAGTGTCGCTATCAT
Tb927.8.7020 over-expression	
Forward primer	ACTAGTATGACGCGGGAGGAA
Reverse Primer	GGATCCCTAGAAGGTCCTGCAACGAA
Tb927.6.400 over-expression	
Forward primer	ACTAGTATGCAGGTTGATTGGGGTGCAGTT
Reverse Primer	GGATCCTCACTGCTTATGTATCTTT
Tb927.11.3570 over-expression	
Forward primer	ACTAGTATGTCCACGATCGAACGGGA
Reverse Primer	GGATCCCTATAACGCTGCAACCGAAA
Tb927.11.6590 over-expression	
Forward primer	ACTAGTATGCCTACTTTACCCAAGGCCGAA
Reverse Primer	GGATCCCTACAATTTTCGGAAGTACT
Tb927.11.2500 over-expression	
Forward	ACTAGTATGAAGGCATACAAAGAGCTCGA
Reverse	GGATCCTCAGTTGGCATCGTCACGGT
Tb927.11.12850 over-expression	
Forward	ACTAGTATGCAAACTGAACGTGGTCC
Reverse	GGATCCCTACTTCCGCAGCAGCTGC
Tb927.3.4750 over-expression	
Forward	ACTAGTATGCCAACTCTCCCCTCAGA
Reverse	GGATCCTCACCGCGGAAAGAACAGG

Supplementary Table 5: List of plasmids used in this study

Plasmid	
pPOTV6-blast-blat-mNeonGreen	Tagging and knockout
pPOTv7-hygro-hygromNeonGreen	Knockout
pDex577-Y	Ectopic overexpression

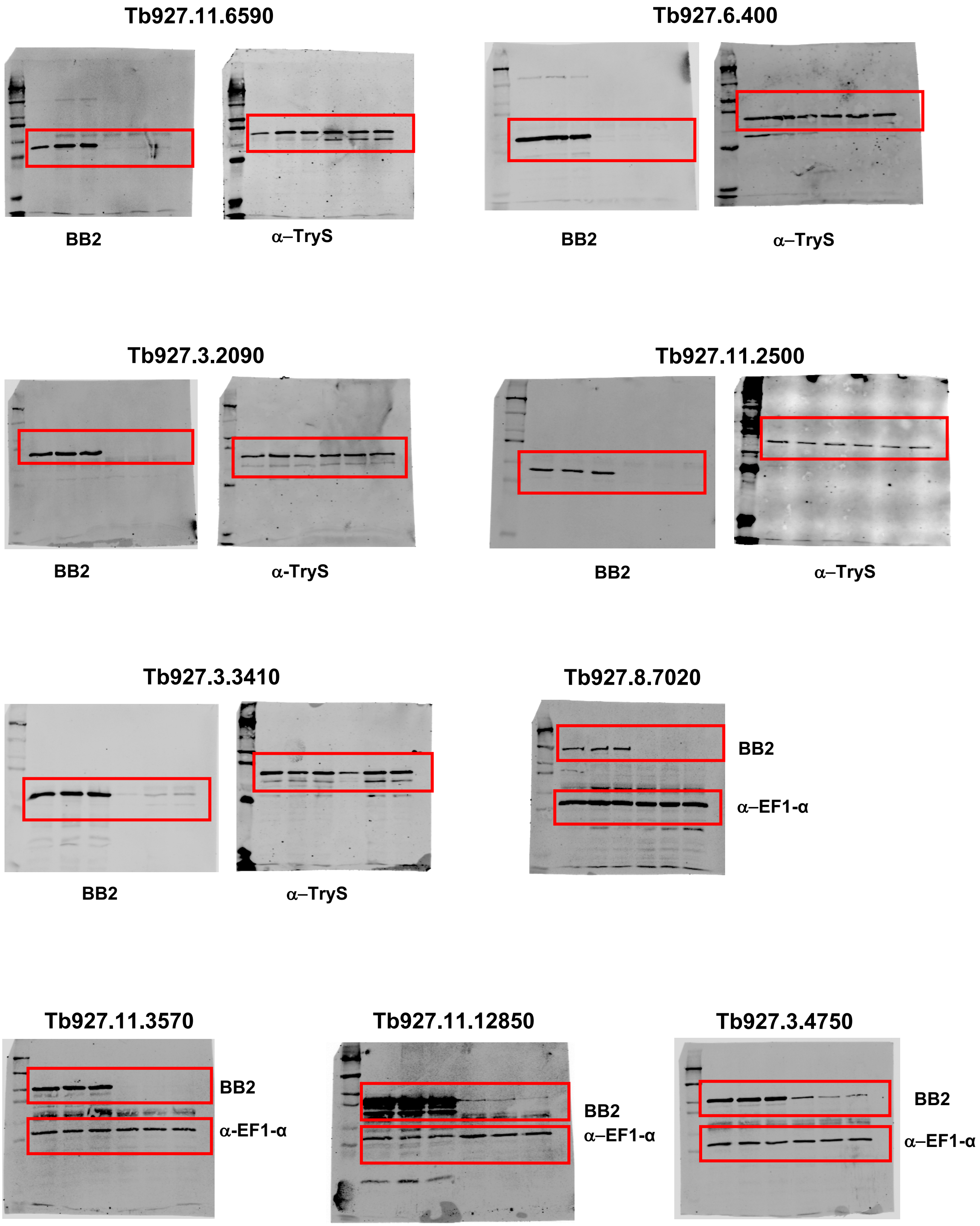

Full length gel images from Supplementary figure 2a, b, c & d. Cropped areas correspond to the released peptidase in the supernatant.

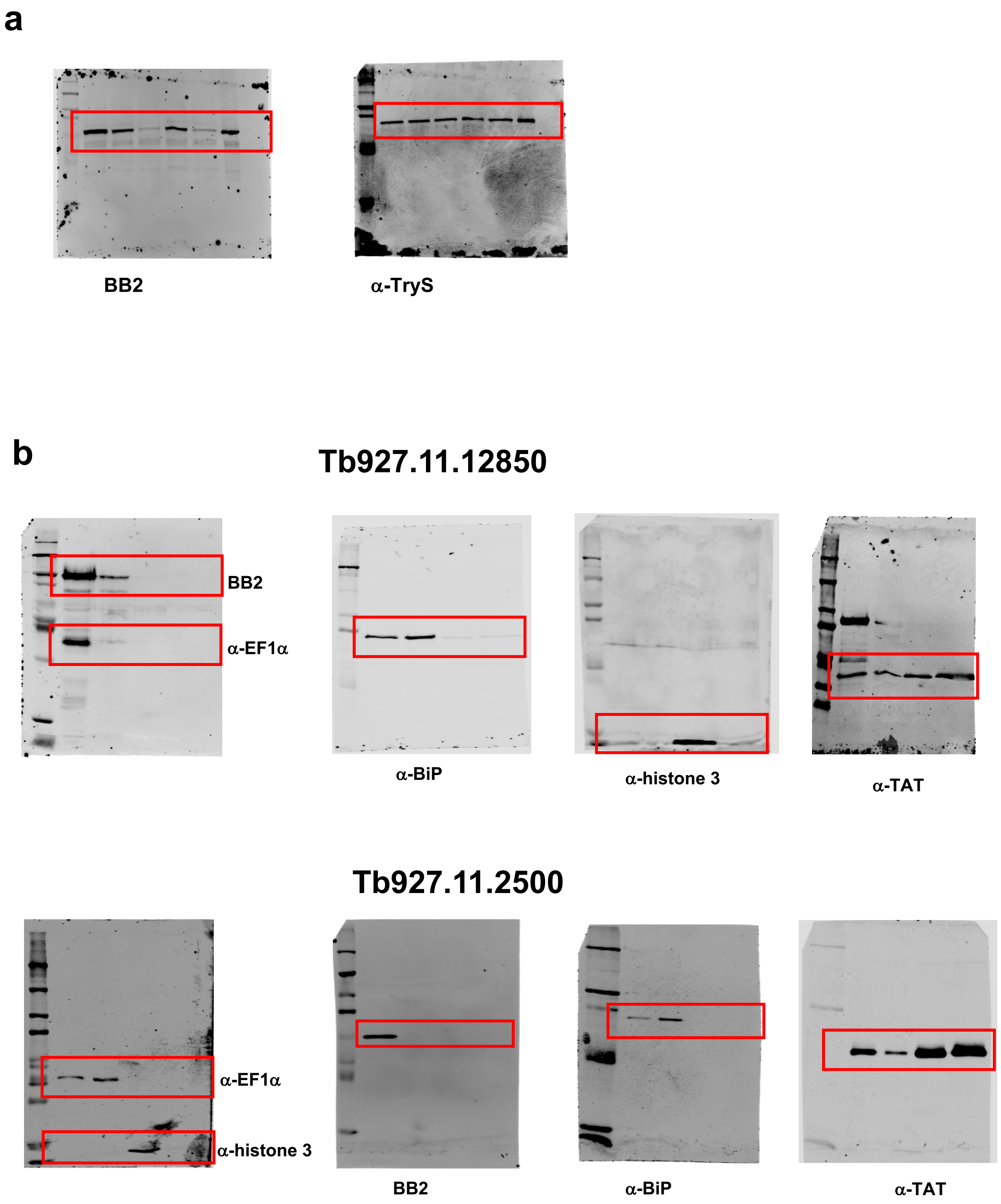

Full length blots from;

a. Supplementary figure 5b
b. Supplementary figure 6
