# Supplementary material for: Extracellular release of two peptidases dominates generation of the trypanosome quorum-sensing signal
Source: Nat Commun. 2022 Jun 9;13:3322. doi: 10.1038/s41467-022-31057-1 (PMC9184580; doi:10.1038/s41467-022-31057-1)
Supplement: Supplementary file 3 — Description of Additional Supplementary Information [file 41467_2022_31057_MOESM3_ESM.docx]

**Description of additional supplementary files**

**File name: Supplementary Data 1**

Description: All the proteins identified to be released by the different developmental stages of the parasite.

**File name: Supplementary Data 2**

Description: Quantification of the identified proteins by progenesis

**File name: Source data files.zip**

Description: a zip file comprising two files:

- **File name**: source data file-uncropped gels.pdf

Description: Full length gel blots for Figure 2, 4, 5 and 7

- **File name**: source date file- graphs.xlsx

Description: Source data for Figure 3-7 and Supplementary Figure 2-7.
