# Supplementary material for: Extracellular release of two peptidases dominates generation of the trypanosome quorum-sensing signal
Source: Nat Commun. 2022 Jun 9;13:3322. doi: 10.1038/s41467-022-31057-1 (PMC9184580; doi:10.1038/s41467-022-31057-1)
Supplement: Supplementary file 6 — Reporting Summary [file 41467_2022_31057_MOESM6_ESM.pdf]

nature portfolio

Corresponding author(s): Keith R. Matthews
Federico Rojas

Last updated by author(s): 26-04-2022

Reporting Summary

Nature Portfolio wishes to improve the reproducibility of the work that we publish. This form provides structure for consistency and transparency in reporting. For further information on Nature Portfolio policies, see our Editorial Policies and the Editorial Policy Checklist.

Statistics

For all statistical analyses, confirm that the following items are present in the figure legend, table legend, main text, or Methods section.

n/a	Confirmed	
[ ]	[x]	The exact sample size (n) for each experimental group/condition, given as a discrete number and unit of measurement
[ ]	[x]	A statement on whether measurements were taken from distinct samples or whether the same sample was measured repeatedly
[ ]	[x]	The statistical test(s) used AND whether they are one- or two-sided<br>*Only common tests should be described solely by name; describe more complex techniques in the Methods section.*
[x]	[ ]	A description of all covariates tested
[ ]	[x]	A description of any assumptions or corrections, such as tests of normality and adjustment for multiple comparisons
[ ]	[x]	A full description of the statistical parameters including central tendency (e.g. means) or other basic estimates (e.g. regression coefficient) AND variation (e.g. standard deviation) or associated estimates of uncertainty (e.g. confidence intervals)
[ ]	[x]	For null hypothesis testing, the test statistic (e.g. F, t, r) with confidence intervals, effect sizes, degrees of freedom and P value noted<br>*Give P values as exact values whenever suitable.*
[x]	[ ]	For Bayesian analysis, information on the choice of priors and Markov chain Monte Carlo settings
[x]	[ ]	For hierarchical and complex designs, identification of the appropriate level for tests and full reporting of outcomes
[x]	[ ]	Estimates of effect sizes (e.g. Cohen's d, Pearson's r), indicating how they were calculated

Our web collection on statistics for biologists contains articles on many of the points above.

Software and code

Policy information about availability of computer code

Data collection	QCapture Suite Plus Software (version 3.1.3.10, https://www.qimaging.com); Gene-specific primers for the amplification of the pPOT plasmid and the small guide RNA (sgRNA) scaffold (G00) were designed using the program at (www.leishgedit.net/Home.html)
Data analysis	Most analyses were carried out in Graphpad Prism version 9<br>(GraphPad Software, La Jolla, California, USA, www.graphpad.com).<br>QCapture Suite Plus Software, version 3.1.3.10<br>(https://www.qimaging.com)<br>ImageJ version 1.53i<br>(Schneider et al., 2012)

For manuscripts utilizing custom algorithms or software that are central to the research but not yet described in published literature, software must be made available to editors and reviewers. We strongly encourage code deposition in a community repository (e.g. GitHub). See the Nature Portfolio guidelines for submitting code & software for further information.

Data

Policy information about availability of data

All manuscripts must include a data availability statement. This statement should provide the following information, where applicable:

- Accession codes, unique identifiers, or web links for publicly available datasets
- A description of any restrictions on data availability
- For clinical datasets or third party data, please ensure that the statement adheres to our policy

The mass spectrometry proteomics data generated in this study have been deposited to the ProteomeXchange Consortium via the PRIDE 36partner repository with

the dataset identifier PXD032101 and 10.6019/PXD032101.
The data comprising parasitaemia scores and gel images are provided with this paper in the accompanying source data files and supplementary information. All biological reagents are available upon request subject to availability or regulatory approval

Field-specific reporting

Please select the one below that is the best fit for your research. If you are not sure, read the appropriate sections before making your selection.

- [x] Life sciences
- [ ] Behavioural & social sciences
- [ ] Ecological, evolutionary & environmental sciences

For a reference copy of the document with all sections, see nature.com/documents/nr-reporting-summary-flat.pdf

Life sciences study design

All studies must disclose on these points even when the disclosure is negative.

Sample size	For the analysis of phenotypes 3- 5 animals per treatment were routinely used for analysis. In previous analyses (e.g. Mony, B.M., et al., Genome-wide dissection of the quorum sensing signalling pathway in Trypanosoma brucei. Nature, 2014. 505(7485): p. 681-5) we showed that this sample size is sufficient to detect differences between cell lines and treatment groups (for example where gene silencing is activated by provision of doxycycline). In that study, we tested 5 genes for effects with and without doxycycline mediated gene-silencing in vivo. Using cell cycle status as the measured parameter, the effect size ranged from 0.637 to 1.804. Those values were then used to calculate the power for different samples sizes. This showed that a sample size of 3-5 per group (+ or - DOX) , or total of 6 to 10 allowed us to achieve 80% power for all test genes except one. In most cases observed phenotypes were detected in pilot studies in addition to the presented datasets.
Data exclusions	An outlier resulting from an atypically poor infection was excluded from the statistical analyses in Figure 4D since it generated n=2. The parasitaemia values remain included in the source data file for information.
Replication	All figures include information on the replicate number for each experiment. Experiments were also validated in additional pilot experiments or independent replicates.
Randomization	Animals were allocated at random into treatment groups from a group of female, age matched MF1 mice.
Blinding	No blinding was done to allow appropriate treatment of the animals e.g. doxycycline provision, health monitoring etc. as required by our UK Home office licencing

Reporting for specific materials, systems and methods

We require information from authors about some types of materials, experimental systems and methods used in many studies. Here, indicate whether each material, system or method listed is relevant to your study. If you are not sure if a list item applies to your research, read the appropriate section before selecting a response.

Materials & experimental systems

n/a	Involved in the study	
[ ]	[x]	Antibodies
[ ]	[x]	Eukaryotic cell lines
[x]	[ ]	Palaeontology and archaeology
[ ]	[x]	Animals and other organisms
[x]	[ ]	Human research participants
[x]	[ ]	Clinical data
[x]	[ ]	Dual use research of concern

Methods

n/a	Involved in the study	
[x]	[ ]	ChIP-seq
[x]	[ ]	Flow cytometry
[x]	[ ]	MRI-based neuroimaging

Antibodies

Antibodies used	anti-Ty1 epitope tag antibody BB2: Bastin et al., 1996; hybridoma cell line a gift of Keith Gull, Oxford University/available through Thermofisher; Cat#MA5-23513; RRID:AB_2610644; used at 1:5<br>Anti-PAD1, Dean et al., 2009; generated in our laboratory. used at 1:1000.<br>Anti-EF1 alpha; Merck Millipore Cat#05-235. used at 1:7000.<br>Anti-rabbit (goat anti-rabbit IgG (H+L) Dylight 800; Thermofisher Cat#SA5-10036; used at 1:5000.
Validation	anti-Ty1 epitope tag antibody BB2: Validated for use as an epitope tag antibody in trypanosomes in Bastin et al., 1996; hybridoma cell line a gift of Keith Gull, Oxford University/available through Thermofisher; Cat#MA5-23513<br>anti-PAD1, validated as a marker for bloodstream stumpy form trypanosomes in Dean et al., 2009 (our laboratory).<br>Anti-EF1 alpha; Merck Millipore Cat#05-235 validated for use in trypanosomes in Rojas et al 2019 (our laboratory)

	Anti-rabbit (goat anti-rabbit IgG (H+L) Dylight 800; Thermofisher Cat#SA5-10036; validated by the manufacturer.

Eukaryotic cell lines

Policy information about cell lines

Cell line source(s)	Trypanosoma brucei EATRO 1125 AnTat1.1 90:13 ; M. Engstler, M. Boshart<br>Cold shock and regulation of surface protein trafficking convey sensitization to inducers of stage differentiation in Trypanosoma brucei Genes Dev., 18 (2004), pp. 2798-2811<br>Trypanosoma brucei EATRO 1125 AnTat1.1 J1339; Rojas et al 2018
Authentication	Molecular validation (inducible expression of integrated target genes or successful gene knockout)
Mycoplasma contamination	Not relevant
Commonly misidentified lines<br>(See ICLAC register)	Not relevant

Animals and other organisms

Policy information about studies involving animals; ARRIVE guidelines recommended for reporting animal research

Laboratory animals	Female MF1 mice older than 6 weeks were used for all experiments and sourced either from Charles River or locally bred.
Wild animals	None used
Field-collected samples	None used
Ethics oversight	All work was carried out under a UK home office licence (P262AE604) that had been approved after local ethical review at the University of Edinburgh Animal Welfare Ethical Review Body and UK Home Office.

Note that full information on the approval of the study protocol must also be provided in the manuscript.
