# Supplementary material for: Extracellular release of two peptidases dominates generation of the trypanosome quorum-sensing signal
Source: Nat Commun. 2022 Jun 9;13:3322. doi: 10.1038/s41467-022-31057-1 (PMC9184580; doi:10.1038/s41467-022-31057-1)
Supplement: Supplementary file 7 — Source data [file 41467_2022_31057_MOESM7_ESM.zip › source data files/Source data file-uncropped gels.pdf]

Extracellular release of two peptidases dominates generation of the trypanosome quorum-sensing signal

Mabel Deladem Tettey, Federico Rojas and Keith R. Matthews

Source data file

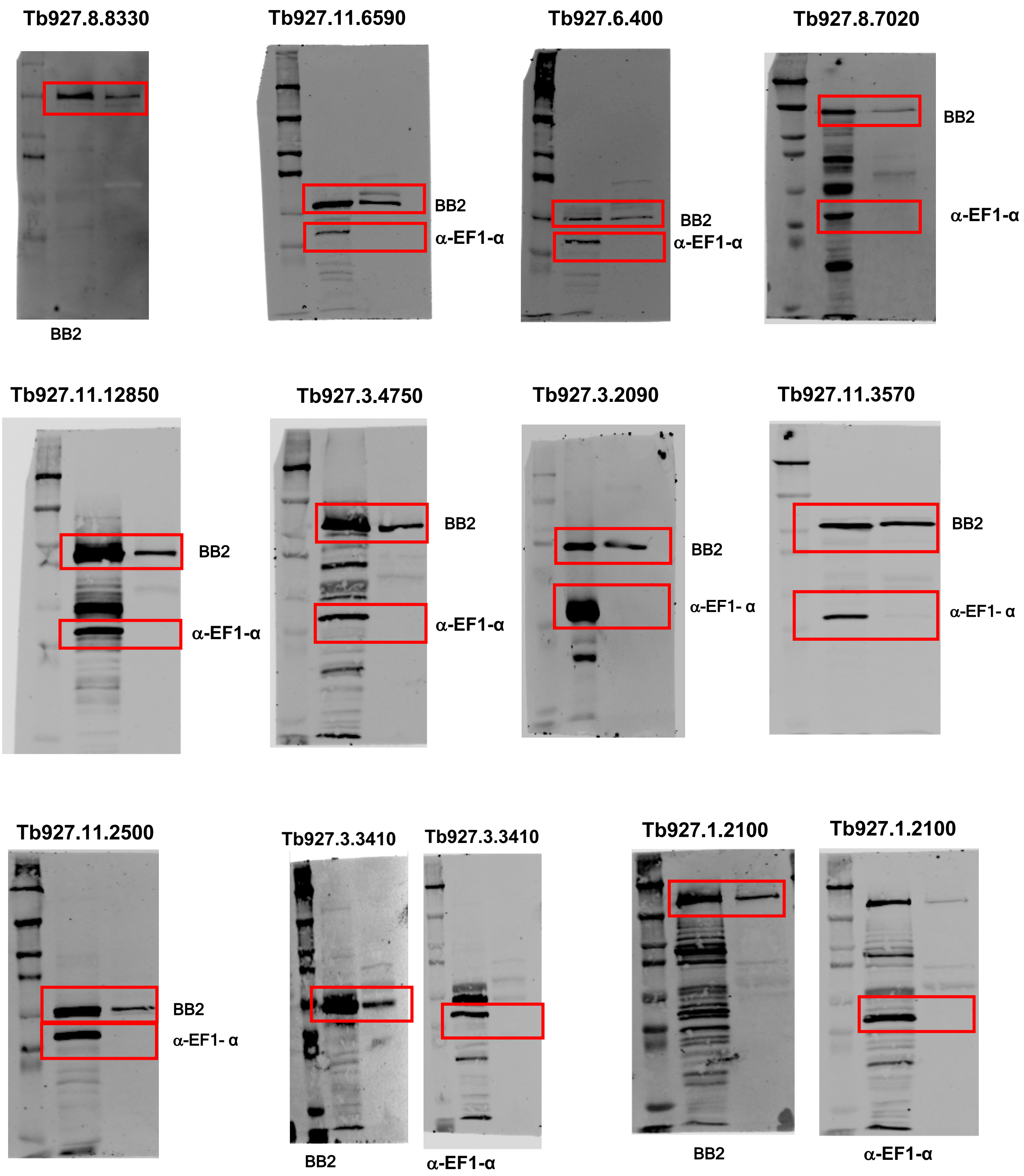

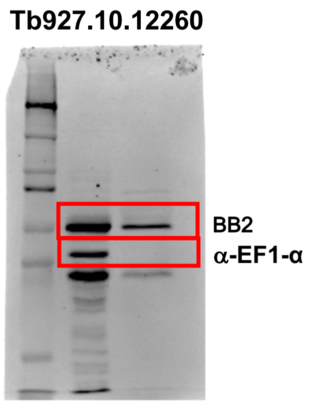

Full length blots from the Figure 2A . Cropped areas correspond to the released peptidase in the supernatant. In the pellet, degradation products were sometimes detected but material released from the cells was intact.

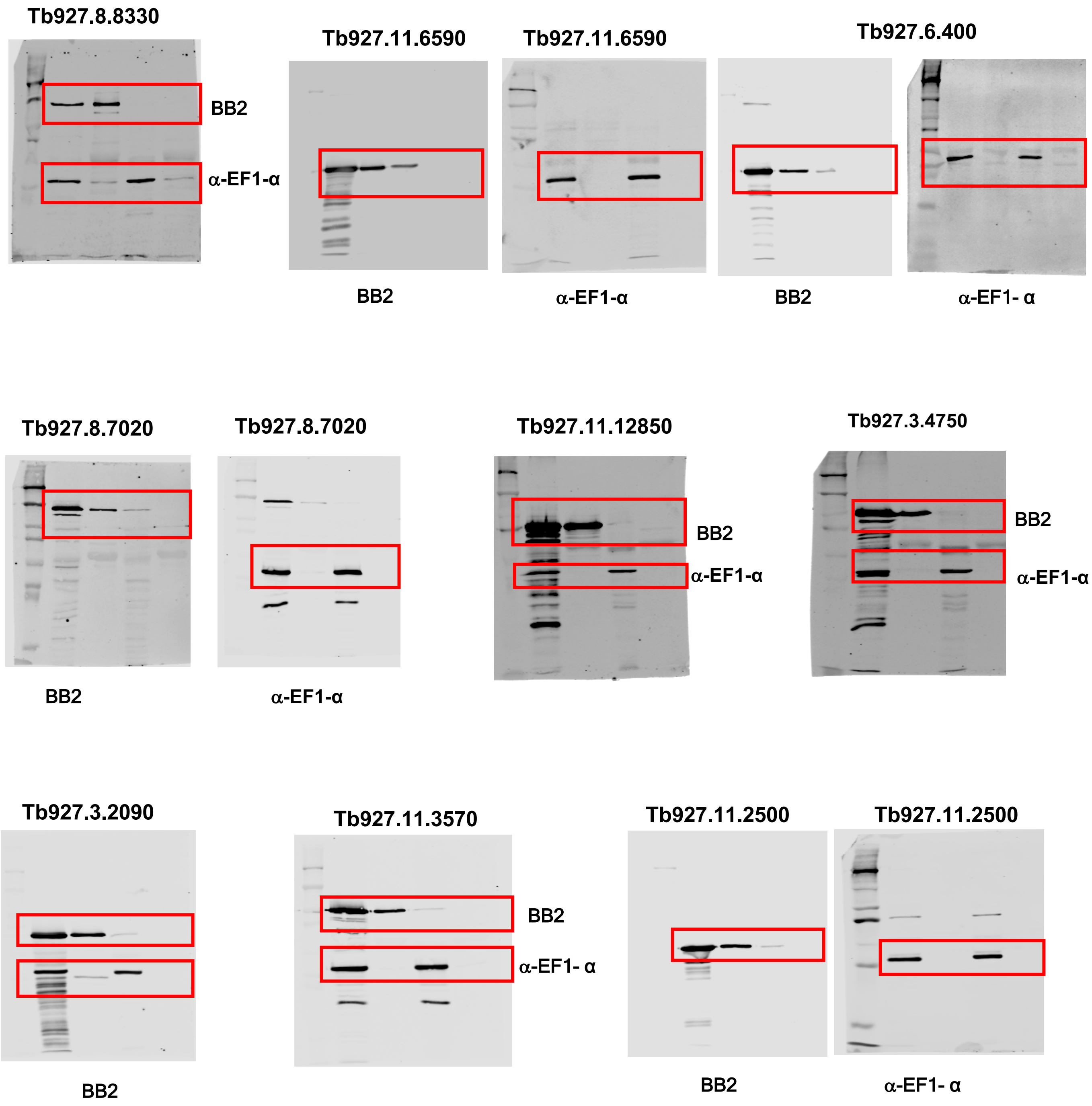

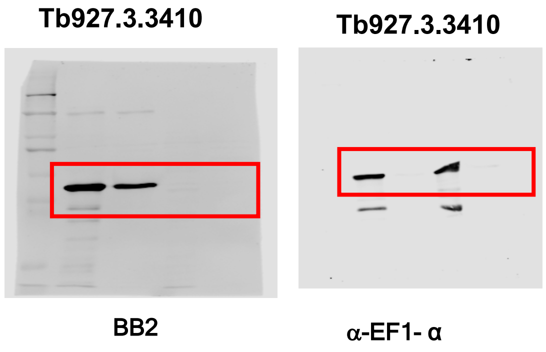

Full length blots for the Figure 2B. Cropped areas correspond to the released peptidase in the supernatant. In the pellet, degradation products were sometimes detected but material released from the cells was intact.

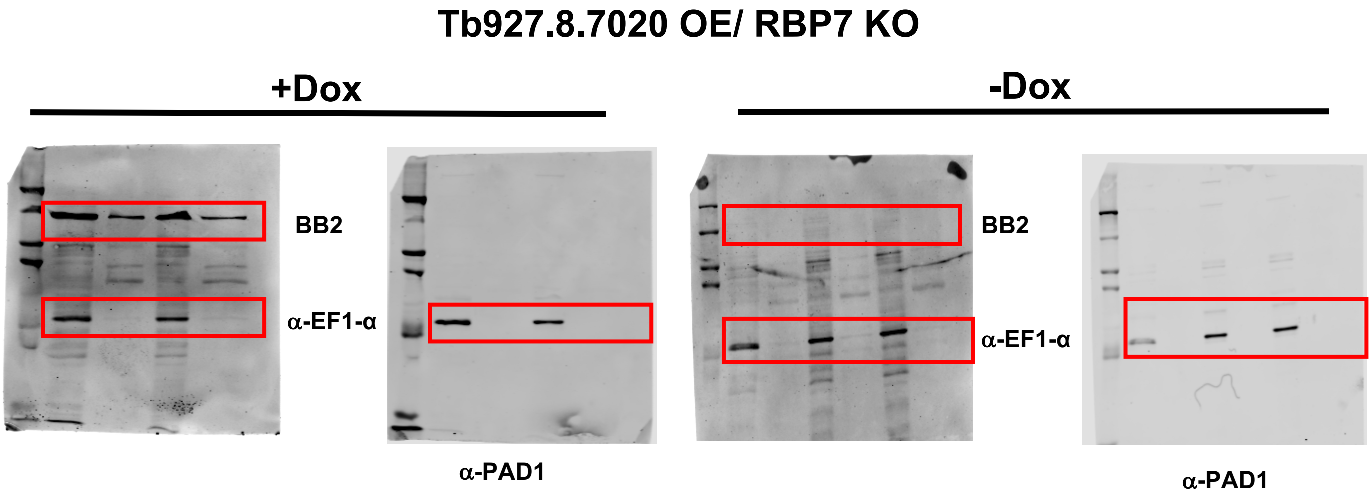

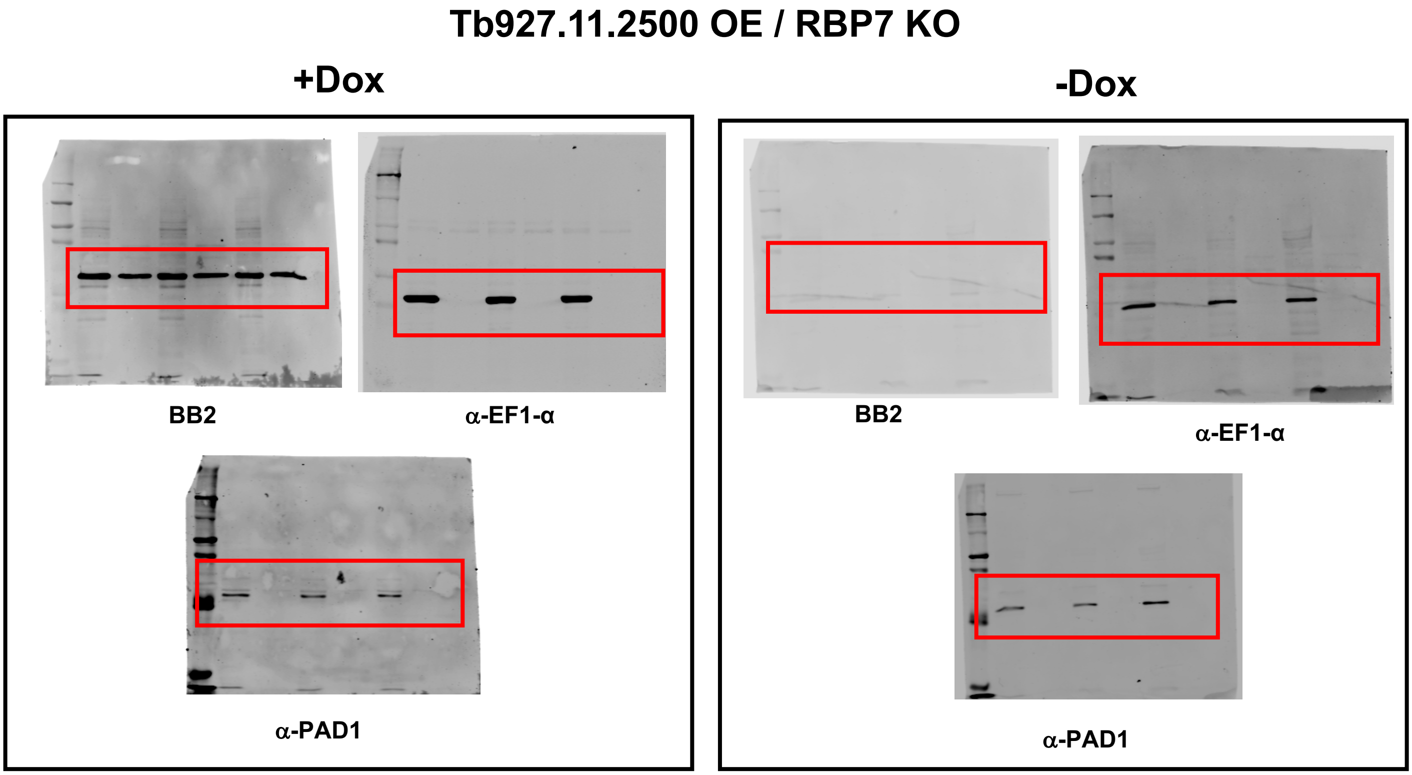

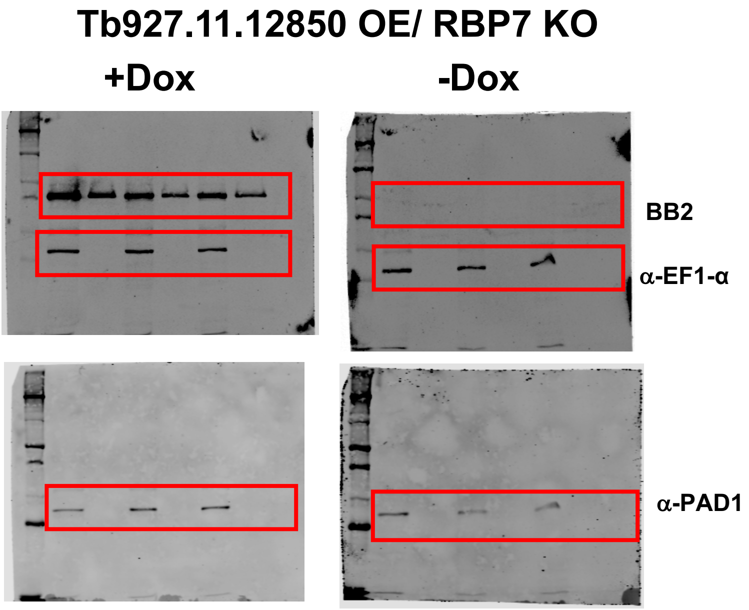

Full length blots from the Figure 4.
Cropped areas correspond to the released peptidase in the supernatant. In the pellet, degradation products were sometimes detected but material released from the cells was intact.

a

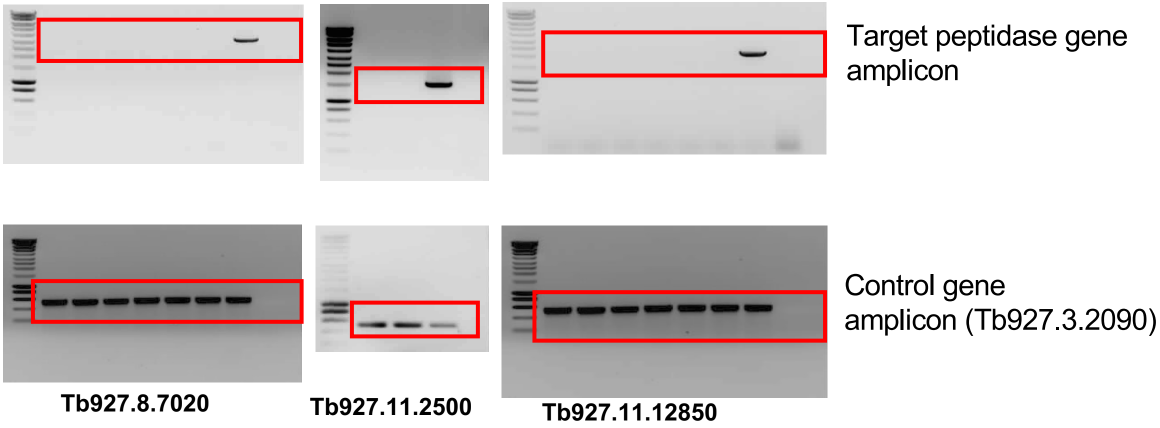

b

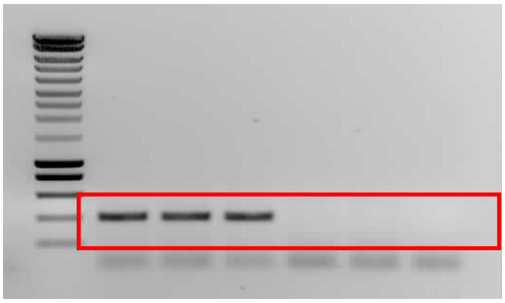

c

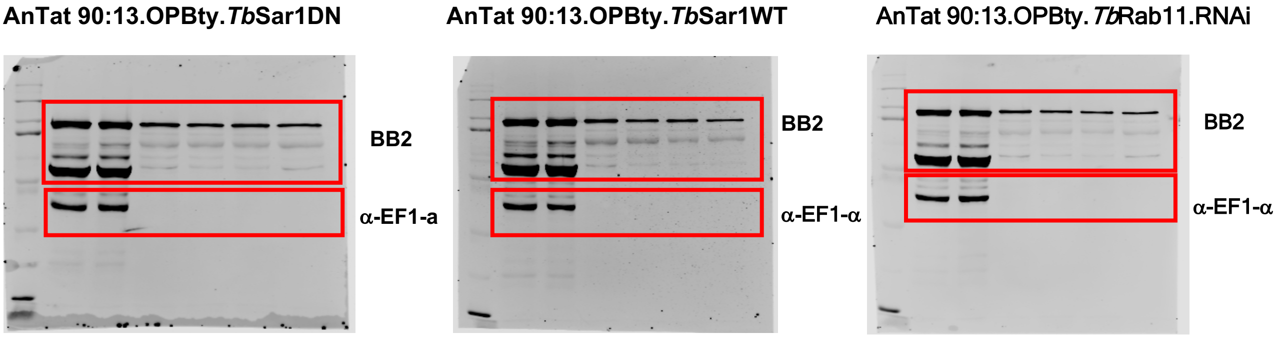

Full length gel and blots from;

a. Figure 5A
b. Figure 5C
c. Figure 7B & D. Cropped areas correspond to the released peptidase in the supernatant; in the pellet a major degradation products was also detected in all samples but this was not seen in the released material.
